# Acetaminophen as Adjuvant to Risperidone in Chronic Schizophrenia: A Randomized, Double-Blind, Placebo-Controlled Clinical Trial 

**Published:** 2018-01

**Authors:** Somaye Arabzadeh, Atefeh Zeinoddini, Seyed-Ali Mostafavi, Mehdi Hamedi, Abolfazl Ehyaii, Ali Ghaleiha, Arefeh Zeinoddini, Shahin Akhondzadeh

**Affiliations:** 1 Psychiatric Research Center, Roozbeh Hospital, Tehran University of Medical Sciences, Tehran, Iran.; 2 Behavioral Disorders and Substance Abuse Research Center, Hamadan University of Medical Sciences, Hamadan, Iran.

**Keywords:** *Acetaminophen*, *Cyclooxygenase*, *Inflammation*, *PANSS*, *Schizophrenia*

## Abstract

**Objective:** Although the pathogenesis of schizophrenia is still uncertain, a variety of predisposing mechanisms have been implicated including inflammatory cascades. The present study was conducted to investigate the effectiveness of acetaminophen as a cyclooxygenase inhibitor in treating patients with schizophrenia.

**Method** **:** A double-blind clinical trial was performed on 52 patients with chronic schizophrenia. Patients received risperidone (up to 6 mg/day) plus either acetaminophen (975mg/day) or placebo. Psychotic symptoms were assessed by the Positive and Negative Syndrome Scale (PANSS) at the onset of the trial, and at 2, 4, 6, and 8 weeks post therapy.

**Results:** Compared to the placebo group, the acetaminophen group showed no significant difference in any subtypes of PANSS. Moreover, the side effect profiles of the 2treatment regimens were not significantly different.

**Conclusion: **Acetaminophen adjuvant to risperidone showed no significant effect in ameliorating symptoms of schizophrenia.

Trial Registration: The trial was registered at the Iranian Registry of Clinical Trials (registration number: IRCT201410251556N67).

Schizophrenia is one of the most severe psychiatric disorders, with a psychopathology that remains uncertain. One theory implicated immunological dysfunctions, such as altered immune cell activities and heightened peripheral and central inflammatory responses in the pathogenesis of schizophrenia (1-3). Evidence of activation of inflammatory processes has been found in postmortem CNS tissue of patients with schizophrenia (4), including changes in cytokine levels and increased cyclooxygenase-2 (COX-2) expression (5, 6). Another line of evidence comes from observations of the therapeutic effects of COX-2 inhibitor medications, such as celecoxib, in patients with schizophrenia. Celecoxib was effective in the treatment of schizophrenia, while tested as an adjuvant agent to different antipsychotics, including risperidone (7, 8), amisulpride (9) and olanzapine (10).

However, the degree of inflammatory cascade involvement and the best choice of anti-inflammatory agents to accompany the antipsychotic medication have remained controversial. The efficacy of celecoxib was inversely correlated to the duration of the disease; the effect was more pronounced in the acute phase of schizophrenia, but was not observed in patients with chronic schizophrenia (7, 9-10). When aspirin, another COX inhibitor, was added to antipsychotics as adjuvant treatment for schizophrenia spectrum disorders, it reduced symptoms of total and positive subscale of PANSS in these patients (12).

Minocycline inhibits cytokine production and reducesCOX-2 expression and prostaglandin E2 production (13, 14).

Two randomized, double-blind, placebo-controlled studies supported minocycline's effect in reduction of positive and negative symptoms of chronic schizophrenia (15, 16).

Acetaminophen is one of the most commonly used antipyretic and analgesic agents worldwide (17). It is absorbed rapidly by the intestinal lumen and metabolized predominantly by the liver with half-life of 2 to 3 hours in adults (18).

Acetaminophen crosses the blood brain barrier easily and is evenly spread throughout the central nervous system, where selectively inhibit both cyclooxygenase-1 (COX-1) and cyclooxygenase-2 (COX-2) enzymes. Hence and acetaminophen predominantly shows its anti-inflammatory and analgesic effects in the brain (19, 20).

Falloon *et al. *conducted a clinical trial to assess the efficacy of acetaminophen in reducing the acute symptoms of schizophrenia and found that acetaminophen was not different from placebo (21). However, acetaminophen was administered to 10 patients for a short period of time, which was not an augmentation therapy. In addition, the patients received haloperidol as their behavior became unmanageable.

Having knowledge about the effects of administering the COX-1 and COX-2 inhibitors in treatment of schizophrenia, we hypothesized that extended administration of acetaminophen may reduce the symptoms of schizophrenia, when used as an adjuvant agent to antipsychotics. To test this hypothesis, we aimed at determining the effects of acetaminophen as adjuvant to risperidone in patients with chronic schizophrenia and exploring the effects of acetaminophen on positive, negative, and general behavioral symptoms of schizophrenia in a randomized, double-blind, placebo-controlled clinical trial.

## Materials and Methods

*Participants*

This was a prospective, 8-week, double-blind study of parallel groups of patients with chronic schizophrenia. The patients were recruited from the pool of patients receiving inpatient treatment at Roozbeh Psychiatric hospital in Tehran, Iran, and at the Department of Psychiatry, Hamadan University of Medical Sciences, Hamadan, Iran, from December 2014 to October 2016. The trial protocol was approved by the Institutional Review Board (IRB) of Tehran University of Medical Sciences (Grant No: 26263) and was conducted in accordance with the Declaration of Helsinki and its subsequent revisions. The trial was registered at the Iranian Registry of Clinical Trials (www.irct.ir; registration number: IRCT201410251556N67). Written informed consent was obtained from all eligible patients and/or their legally authorized representatives. Patients were informed that they were free to withdraw from the study at any time without any influence on their relationship with their healthcare provider.

**Figure 1:**
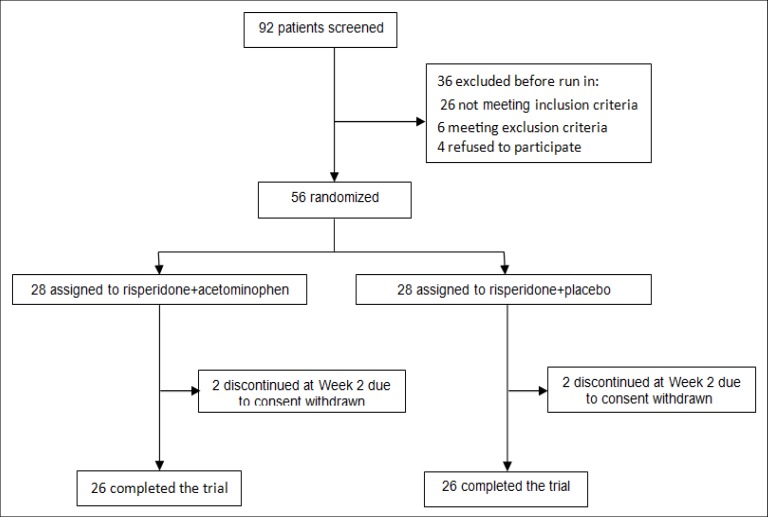
Flowdiagram of the Patients Enrolment in the Study

At the beginning of the study, 92 patients with the diagnosis of chronic schizophrenia were screened. Considering the inclusion and exclusion criteria, 36 candidates were omitted. The remaining 56 patients were randomly assigned into 2groups. During the 8-week follow-up period, 2patients dropped out of each group (Figure 1). 

*Inclusion Criteria*

The inclusion criterion in this study was the diagnosis of schizophrenia using the Structured Clinical Interview for Axis I DSM-V Disorders (SCID).The Persian version of SCID shows kappa of 0.4 (acceptable) for diagnosis of psychiatric disorders (22) and has been used for diagnosis of schizophrenia in multiple previous trials in Iran (8, 23). All participants were inpatients, in the active phase of illness, aged 19 to 54 years, and all met the DSM-V criteria for schizophrenia. At the admission time, patients underwent a physical examination; moreover, blood and urine samples were taken from all the participants; furthermore, complete blood count, alanine aminotransferase (ALT), aspartame aminotransferase (AST), blood urea nitrogen (BUN), and creatinine(Cr) were also checked in all participants. All patients were free of any infection and autoimmune disease for at least 2weeks before entering the trial, they had normal complete blood counts, hepatic, and renal panels, and negative toxicological screens. A minimum score of 60 on the Positive and Negative Syndrome Scale (PANSS) was required to enter the study (24). Patients did not receive neuroleptics from 1week prior to entering into the trial or depot neuroleptic at least 2months before the study. Two senior residents of psychiatry performed the structured clinical interview for DSM-V (SCID) in patients’ native language, Farsi. Eligible participants in the study were 56 patients with chronic schizophrenia.

*Exclusion Criteria*

Patients were excluded from the study if they had a clinically significant organic or neurological disorder identified by a thorough physical examination, drug abuse, or dependence within the past 6 months, or any psychotic disorder other than schizophrenia. Pregnant or lactating women and those of reproductive age without adequate contraception were also excluded. Patients with significant depression, defined as a score≥14 on the 17-item Hamilton Depression Rating Scale (HDRS) (Hamilton 1960), or a score of ≥ 4 on the depression item of PANSS were excluded from the study. Patients were also excluded if they had received electroconvulsive therapy (ECT) during the last 2weeks prior to entering the trial. Patients did not use antidepressants, mood stabilizers, or a second antipsychotic (as an augmentative strategy) during the course of the trial.

*Intervention*

Patients were randomly allocated into 2 groups: 28 patients were allocated to the risperidone (6 mg/day) plus acetaminophen (975 mg/day; 325 mg t.i.d) group, and 28 patients were allocated to the risperidone (6 mg/day) plus placebo group for an 8-week therapy. Starting dosage of risperidone was 2 mg/day and was increased, with 2 mg increments, to 6 mg/day daily dosage for the first 2days. Patients in the placebo group received 3identical capsules a day. Patients also received biperiden if they showed extrapyramidal symptoms. Patients were assessed by a psychiatrist at baseline and at 2, 4, 6, and 8 weeks after medication administration.

*Outcome*

The principal measure of the outcome was the Positive and Negative Syndrome Scale (PANSS).This instrument is a standard scale for measuring severity of symptoms of patients with schizophrenia. The rater used standardized instructions when using the PANSS (24). Reliability and validity of the PANSS scale for patients with schizophrenia have been approved by its inventor with high internal consistency and inverse correlation of positive and negative symptoms (24); the alpha Cronbach for this instrument in Persian studies has been reported to be between 73% to 83%(25). The mean decrease in PANSS score from baseline was used as the main outcome measure of response to treatment. 

The extrapyramidal symptoms were assessed using the extra pyramidal Symptoms Rating Scale (ESRS) (part one: Parkinsonism, dystonia, dyskinesia, and sum of 11 items). The ESRS was created to evaluate 4 types of drug-induced movement disorders (DIMD): Parkinsonism, akathisia, dystonia, and tardive dyskinesia (TD); its reliability and validity have been approved by high inter-rater reliability coefficient (80%≤) and concurrent validity of having more than 96% agreement between defined cases by DSM-IV TD criteria (26). PANSS and ESRS have been used in a number of previous trials in Iran (27, 28). PANSS and ESRS were rated by 2 trained senior residents of psychiatry. These tests were performed in patients’ native language, Farsi. Side effects were systematically recorded throughout the study and were assessed using a checklist (29) administered by a resident of psychiatry. All assessments were performed at baseline and at weeks 2, 4, 6 and 8. In addition, HDRS was administered at baseline and Week 8 to assess changes in depressive symptoms. This clinician-rated scale contains 17 questions (measured on either 3- or 5-point scales), which assess the severity of depression-related symptoms; its validity in measuring the symptoms of depression has been approved by Hamilton 1960 (30). The Persian version of HDRS test was validated and applied in a number of previous trials in Iran (31, 32).

*Sample Size*

A sample size of 46 was calculated (23 in each group) assuming to find a final difference of 4 scores on the PANSS rating scale, with type I error of 0.05, and power of 90% (according to the pilot study of this research). Considering an attrition rate of 10%, a final sample size of 50 was needed (25 in each group).

Randomization, Allocation, Concealment, and Blinding

Patients were randomized to receive acetaminophen or placebo in a 1:1 ratio using a computer-generated code. Throughout the study, the person, who administrated the medications, the rater, and the patients were blind to assignments.

*Statistical Analysis*

A two-way repeated measures analysis of variance (time×treatment interaction) was used. The two groups were considered as a between-subjects factor (group) and the5measurements during the treatment were considered as the within-subjects factor (time). This was done for positive, negative, general psychopathology subscale, and PANSS total scores. A Greenhouse–Geisser correction was used for sphericity. An unpaired student's t test with a two-sided p- value was used to compare the 2 groups at baseline and to determine the outcome of the 2groups at the end of the trial. Fisher's exact test was performed to compare the demographic data and frequency of side effects between protocols. Results are presented as mean±SEM. Statistical significance was set at p <0.05. Intention to treat (ITT) analysis, with last observation carried forward (LOCF) procedure, was performed. Statistical analysis was performed using Statistical Package of Social Science Software (SPSS Version 20, IBM Company and USA). Sigma plot (version 12) was used to draw the graphs of repeated measure tests.

## Results

*Demographic Data*

A total of 26 participants aged 19 to 54finished the study in each group. There were no significant differences in baseline demographic data between the 2groups (acetaminophen vs. placebo; Table 1).

*PANSS Positive Subscale*

**Figure 2:**
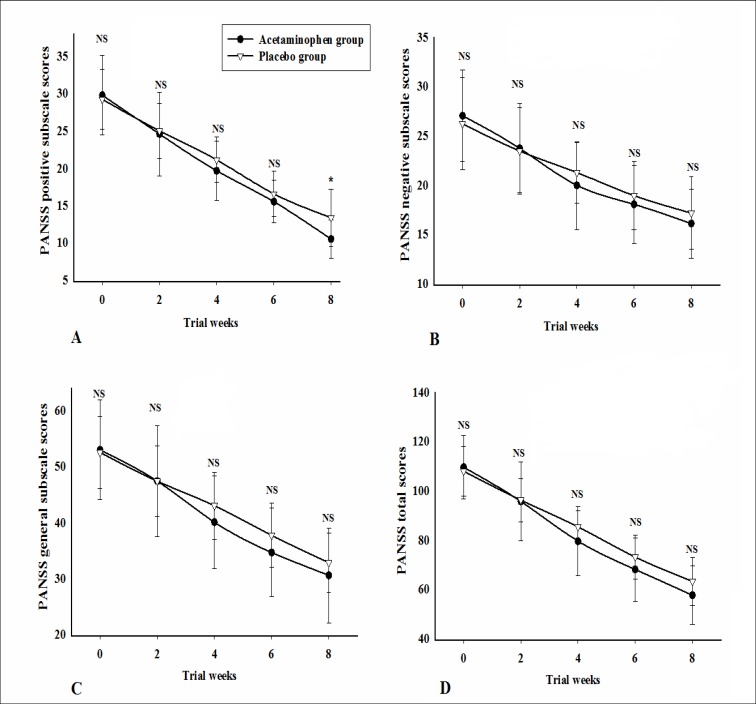
Repeated Measure for Comparison of the Effects of the Two Treatments on the Positive and Negative Syndrome Scale (PANSS),Positive (A), Negative (B), General Psychopathology (C), and Total (D) Subscales Scores

Baseline PANSS positive subscale scores were not significantly different between the 2 groups(Table 1).General linear model repeated measures demonstrated no significant effect for time × treatment interaction on PANSS positive subscale scores [F (1.83, 91.70) = 2.40, P = 0.10] (Figure 2A). Comparison of PANSS positive subscale scores showed no significant differences between the 2groups, except at Week 8(Table 2). 

*PANSS Negative Subscale*

Baseline PANSS negative subscale scores were not significantly different between the 2 groups (Table 1).General linear model repeated measures demonstrated a significant effect for time × treatment interaction on PANSS negative subscale scores[F (2.14, 107.41) = 2.25, P =0.10] (Figure 2B). Comparison of PANSS negative subscale scores revealed no significant difference between the 2groups (Table 2). 

*PANSS General Psychopathology Subscale*

Baseline PANSS general psychopathology scores were not significantly different between the 2groups (Table 1).General linear model repeated measures demonstrated no significant effect for time × treatment interaction on PANSS general psychopathology subscales cores [F (1.74, 87.44) =1.83, P = 0.17] (Figure 2C). Comparison of PANSS general psychopathology subscale scores revealed no significant difference between the 2groups (Table 2).

*PANSS Total Score*

Baseline PANSS total scores were not significantly different between the 2groups (Table 1).General linear model repeated measures demonstrated no significant effect for time × treatment interaction on PANSS total scores [F (1.65, 82.86) =3.09, P = 0.60] (Figure 2D). Comparison of PANSS total scores showed no significant difference between the two groups (Table 2).

*Hamilton Depression Rating Scale (HDRS)*

Baseline Hamilton Depression Rating Scale (HDRS) scores were not significantly different between the two groups (Table 1). Baseline mean HDRS scores were below 14 in both groups, which indicated no significant depressive symptoms in patients. No significant difference was observed between the2 treatment groups on reduction of HRDS scores from baseline to the study end point [MD (95% CI) =− 0.15 (−0.55 to 0.24), t (50) = − 0.77, P = 0.44].

*Extrapyramidal Symptoms Rating Scale (ESRS)*

ESRS scores did not differ significantly between the study groups at baseline (Table 1). No significant effect was observed for time × treatment interaction in the ESRS scores [F (2.73, 136.48) = 1.52, P = 0.21]. The difference was not significant between treatment groups in score change from baseline to the endpoint [MD (95% CI) = −1.84 (−4.12 to 0.43), t (50) =− 1.62, P = 0.11].

*Adverse Events*

No deaths or serious adverse events requiring medical intervention were reported. According to the adverse events checklist, there was no significant difference between the 2groups in the frequency of adverse events (Table 3).

**Table 1:** Baseline Characteristics of the Participants in the Study of Acetaminophen and Risperidone in Chronic Schizophrenia

**Variable**	**Acetaminophen** **(N / ** **mean ± SD** **)**	**Placebo** **(N / ** **mean ± SD** **)**	**P- value**
Gender Female Male	10 16	12 14	ns
Age (year)	32.00 ± 7.05	33.04 ± 9.52	ns
Marital status Single Married Divorced	16 8 2	15 9 2	ns
Level of education Illiterate Primary school High school diploma University degree	1 12 8 5	1 11 7 7	ns
Smoking, n,	22	23	ns
Duration of illness (years)	78.54 ± 41.61	80.38 ± 46.61	ns
Type of schizophrenia Paranoid Residual Disorganized Undifferentiated	14 4 4 4	15 3 4 4	ns
Prior antipsychotic medications Risperidone Halopridol Fluphenazine Olanzapine	15 8 6 9	16 9 6 10	ns
Baseline scores PANSS total score PANSS positive subscale PANSS negative subscale PANSS general psychopathology subscale HDRS ESRS	109.58 ± 12.76 29.77 ± 5.33 27.04 ± 4.64 52.96 ± 8.88 8.08 ± 1.78 2.50± 4.65	107.96 ± 10.10 29.33 ± 4.04 26.23 ± 4.66 52.50 ± 6.39 7.46 ± 1.72 1.62± 2.95	ns ns ns ns ns ns

SD, standard deviation; PANSS, Positive and Negative Syndrome Scale; HDRS, Hamilton Depression Rating Scale; ESRS, Extrapyramidal Symptom Rating Scale; N, number; ns, not significant

**Table 2:** Comparison of PANSS Total and Subscale Scores in the Treatment Groups

**Measurement**	**Acetaminophen + ** **risperidone** **(mean ± SD)**	**Placebo + ** **risperidone** **(mean ± SD)**	**t (50)**	**P- value**	**Cohen’s d**
PANSS total score Week 2 Week 4 Week 6 Week 8	95.69 ± 16.06 79.54 ± 14.06 68.04 ± 12.89 57.58 ± 11.82	96.23 ± 8.77 85.35 ± 6.68 73.08 ± 8.88 63.19 ± 9.81	0.15 1.90 1.64 1.86	0.88 0.06 0.10 0.06	0.04 0.53 0.46 0.52
PANSS positive subscale score Week 2 Week 4 Week 6 Week 8	24.58 ± 5.60 19.65 ± 4.00 15.50 ± 2.84 10.50 ± 2.55	25.00± 3.67 21.12 ± 3.05 16.54 ± 3.04 13.35 ± 3.80	0.32 1.47 1.26 3.16	0.74 0.14 0.21 0.003*	0.09 0.41 0.35 0.89
PANSS negative subscale score Week 2 Week 4 Week 6 Week 8	23.73 ± 4.53 19.96 ± 4.46 18.04 ± 3.96 16.08 ± 3.50	23.46 ± 4.37 21.27 ± 3.08 18.92 ± 3.45 17.15 ± 3.64	-0.21 1.22 0.85 1.08	0.82 0.22 0.39 0.28	-0.06 0.34 0.24 0.30
PANSS general psychopathology subscale score Week 2 Week 4 Week 6 Week 8	47.38 ± 9.88 40.04 ± 8.26 34.62 ± 7.93 30.54 ± 8.46	47.38 ± 6.29 43.00± 6.00 37.69 ± 5.74 32.81 ± 5.25	0 1.47 1.60 1.16	1.00 0.14 0.11 0.25	0 0.36 0.45 0.32

PANSS, positive and negative syndrome scale; SD, standard deviation;

* statistically significant

**Table 3:** Frequency of Adverse Events in the Study of Acetaminophen and Risperidone in Chronic Schizophrenia

**Side effect**	**Risperidone + acetaminophen**	**Risperidone + placebo**	**P- Value (Fisher exact test)**
Constipation	2 (7.69%)	0 (0%)	0.490*
Tremor	1 (3.85%)	1 (3.85%)	0.999*
Increased Appetite	2 (7.69%)	2 (7.69%)	0.999*
Loss of appetite	3 (11.54%)	1 (3.85%)	0.610*
Fatigue	1 (3.85%)	3 (11.54%)	0.610*
Dry mouth	4 (15.38%)	6 (23.08%)	0.726*
Sedation	4 (15.38%)	3 (11.54%)	0.999*
Dizziness	5 (19.23%)	4 (15.38%)	0.999*
Vomiting	2 (7.69%)	3 (11.54%)	0.999*
Nausea	1 (3.85%)	2 (7.69%)	0.999*
Headache	1 (3.85%)	2 (7.69%)	0.999*
Abdominal pain	2 (7.69%)	3 (11.54%)	0.999*
Diarrhea	3 (11.54%)	2 (7.69%)	0.999*

* Not Significant

## Discussion

The inflammatory cascades have been implicated in the psychopathology of schizophrenia. The aim of our study was to investigate the effectiveness of acetaminophen as a cyclooxygenase inhibitor in the treatment of patients with schizophrenia. Acetaminophen inhibits both isoforms of cyclooxygenase (COX-1 and COX-2) primarily in the central nervous system, causing an increase in the pain threshold. Acetaminophen shows 4-fold selectivity for COX-2, causing an almost complete inhibition of COX-2 and a moderate inhibition of COX-1 (33). No significant effect of acetaminophen was found in symptoms of schizophrenia as measured by different subscales of PANSS. 

Our results are consistent with a previous study which reported no significant effect of acetaminophen on acute symptoms of schizophrenia (21). Falloon *et al. suggest* that the florid psychotic phenomena may be related to an excess of prostaglandin E in the hypothalamus (34). Therefore, they performed this trial on 10 patients with schizophrenia in a cross-over trial. Administration of acetaminophen (1g q.i.d) for 1 week was not more effective than placebo in resolving acute symptoms of schizophrenia (21).

A review of the literature reveals controversies in the effectiveness of anti-inflammatory agents in general, and COX-2 inhibitors in particular, in treatment of schizophrenia. Muller et al. found a beneficial effect of the COX-2 inhibitor, celecoxib, add-on in the treatment of schizophrenia (9). Rapaport et al. observed no therapeutic effect of celecoxib augmentation on any aspects of psychopathology in continuously ill outpatients with schizophrenia (11). The study of Rapaport *et al. *involved a group of outpatients with chronic schizophrenia, while Muller et al. investigated in patients with acute exacerbation of the disease. It is possible that celecoxib augmentation potentiates the speed of response, but not the overall magnitude of response. Rapaport concluded that the benefit of celecoxib augmentation for those with an acute psychotic exacerbation cannot be extended to continuously symptomatic outpatients with schizophrenia (11). Contrary to this conclusion, Akhondzadeh et al. showed the effectiveness of celecoxib add-on to risperidone in attenuating positive and general psychopathology, as well as PANSS total score on a group of patients with chronic schizophrenia (8).

This controversy is also reflected in Sommer *et a*.s’ review of anti-inflammatory agents in treatment of schizophrenia. Five studies that administered celecoxib as a COX-2 inhibitor produced heterogeneous results varying from strong positive effect to strong negative effect. The overall analysis of the data revealed an effect size of 0.15, which was not statistically significant (35). Consistent with this review, Yokoto et al. found that COX-2wasnot involved in pathophysiology of schizophrenia. Immunohistochemical analysis of neuronal COX-2 expression in the hippocampus showed no up- regulation of COX2in patients with schizophrenia (36). This finding suggests that any potential effect of celecoxib in attenuating symptoms of schizophrenia may be via COX-2 independent pathways.

Like acetaminophen, aspirinaffectsCOX-1 and COX-2.However, aspirin’s effect on COX-1 is irreversible and it does not pass the blood brain barrier. Two previous studies in the literature used aspirin as an adjuvant therapy for conventional treatment of patients with chronic schizophrenia. The accumulative results showed an effect size of 0.3, which was statistically significant (35). Accumulative evidence on therapeutic effects of different anti-inflammatory agents in patients with schizophrenia showed potential positive effects of aspirin, N-acetyl cysteine and estrogens, when added to antipsychotic treatment. However, as these agents are all broadly active substances, the beneficial effects on symptom severity may not be mediated by their anti-inflammatory aspects (11).

Values represent mean ± SD. NS: non-significant. 

## Limitation

One limitation of the current study was that it included a sample of inpatients only. Future studies with a larger number of inpatients as well as outpatients can help generalize the findings. Another limitation of our study was the lack of measurement of biological parameters, such as IL-2, IL-6, TNF-alpha, and toxoplasma gondii antibodies, as quantitative indicators of inflammatory cascade. Future follow- up studies that measure these biomarkers could better characterize the effect of acetaminophen adjuvant used for the patient group.

## Conclusion

This study revealed no significant role for acetaminophen as adjunct to risperidone in the treatment of patients with schizophrenia. This finding adds to the controversy around the use of COX-2 inhibitor agents for treatment of schizophrenia. Future studies with larger number of patients that are combined with a basic immunochemistry assessment may help resolve this controversy.
